# Supplementary material for: Role and mechanism of NCAPD3 in promoting malignant behaviors in gastric cancer
Source: Front Pharmacol. 2024 Apr 22;15:1341039. doi: 10.3389/fphar.2024.1341039 (PMC11070777; doi:10.3389/fphar.2024.1341039)
Supplement: Supplementary file 11 [file DataSheet2.ZIP › GSEA/Transcipt factor/my_analysis.Gsea.1599461814734/P53_02.html]

Details for gene set P53\_02[GSEA]

|  || Dataset | filtered\_dataset.sample\_info.cls#WT\_versus\_NCAPD3\_MUT |
| Phenotype | sample\_info.cls#WT\_versus\_NCAPD3\_MUT |
| Upregulated in class | NCAPD3\_MUT |
| GeneSet | P53\_02 |
| Enrichment Score (ES) | -0.52572006 |
| Normalized Enrichment Score (NES) | -2.572138 |
| Nominal p-value | 0.0 |
| FDR q-value | 0.0017583675 |
| FWER p-Value | 0.004 |
Table: GSEA Results Summary

  

Fig 1: Enrichment plot: P53\_02      
 Profile of the Running ES Score & Positions of GeneSet Members on the Rank Ordered List

  

| SYMBOL | TITLE | RANK IN GENE LIST | RANK METRIC SCORE | RUNNING ES | CORE ENRICHMENT || 1 | 659 | BMPR2 | 16 | 1.047 | 0.0581 | No |
| 2 | 4193 | MDM2 | 131 | 0.769 | 0.0271 | No |
| 3 | 6507 | SLC1A3 | 464 | 0.496 | -0.1793 | No |
| 4 | 10478 | SLC25A17 | 525 | 0.460 | -0.1919 | No |
| 5 | 149076 | ZNF362 | 680 | 0.388 | -0.2772 | No |
| 6 | 8904 | CPNE1 | 814 | 0.308 | -0.3526 | No |
| 7 | 8848 | TSC22D1 | 899 | -0.324 | -0.3916 | No |
| 8 | 4616 | GADD45B | 904 | -0.330 | -0.3725 | No |
| 9 | 301 | ANXA1 | 914 | -0.342 | -0.3562 | No |
| 10 | 9414 | TJP2 | 916 | -0.345 | -0.3339 | No |
| 11 | 6430 | SRSF5 | 1183 | -0.553 | -0.4889 | Yes |
| 12 | 3866 | KRT15 | 1214 | -0.589 | -0.4713 | Yes |
| 13 | 10150 | MBNL2 | 1217 | -0.591 | -0.4335 | Yes |
| 14 | 8061 | FOSL1 | 1222 | -0.597 | -0.3966 | Yes |
| 15 | 1956 | EGFR | 1266 | -0.657 | -0.3839 | Yes |
| 16 | 91947 | ARRDC4 | 1282 | -0.671 | -0.3500 | Yes |
| 17 | 3084 | NRG1 | 1320 | -0.739 | -0.3275 | Yes |
| 18 | 6646 | SOAT1 | 1333 | -0.755 | -0.2859 | Yes |
| 19 | 56605 | ERO1B | 1340 | -0.778 | -0.2385 | Yes |
| 20 | 1649 | DDIT3 | 1341 | -0.779 | -0.1866 | Yes |
| 21 | 51703 | ACSL5 | 1353 | -0.800 | -0.1413 | Yes |
| 22 | 255488 | RNF144B | 1362 | -0.834 | -0.0915 | Yes |
| 23 | 9990 | SLC12A6 | 1379 | -0.922 | -0.0417 | Yes |
| 24 | 23650 | TRIM29 | 1384 | -0.951 | 0.0187 | Yes |
Table: GSEA details [plain text format]

  

Fig 2: P53\_02      
 Blue-Pink O' Gram in the Space of the Analyzed GeneSet

  

Fig 3: P53\_02: Random ES distribution      
 Gene set null distribution of ES for **P53\_02**
